# In utero exposure to antidepressants and the use of drugs for pulmonary diseases in children

**DOI:** 10.1007/s00228-012-1314-6

**Published:** 2012-07-20

**Authors:** P. G. J. ter Horst, H. J. Bos, L. T. W. de Jong-van de Berg, B. Wilffert

**Affiliations:** 1 Department of Clinical Pharmacy, Isala Klinieken, Groot Wezenland 20, 8011 JW Zwolle, The Netherlands; 2 Unit of PharmacoEpidemiology & PharmacoEconomics, Department of Pharmacy, University of Groningen, Groningen, The Netherlands; 3 Unit of Pharmacotherapy & Pharmaceutical Care, Department of Pharmacy, University of Groningen, Groningen, The Netherlands

**Keywords:** Antidepressants, In utero exposure, Asthma, Teratogenicity

## Abstract

**Purpose:**

The use of antidepressants during pregnancy is common. Some studies suggest an association between in utero exposure to antidepressants and the occurrence of pulmonary diseases like asthma later in life. Serotonin reuptake inhibitors (SSRIs) as well tricyclic antidepressants (TCAs) are thought to be involved in the development of the respiratory rhythm generator (RRG) and the maturation of the formation of surfactant. In this study the use of drugs for pulmonary diseases in children who were exposed to antidepressants in utero were compared with non-exposed children.

**Methods:**

The pharmacy prescription database IADB.nl was used for a cohort study in which the use of drugs for pulmonary disease in children after in utero exposure to antidepressants (TCAs, SSRIs) was compared with children with no antidepressant exposure in utero. Drugs for pulmonary diseases were applied as a proxy for disturbed development of the respiratory tract.

**Results:**

A small though significant increase in the incidence risk ratio (IRR) of the use of drugs for pulmonary disease was found after any-time in utero exposure to SSRIs, adjusted for maternal use of antibiotics, of 1.17 (95 % CI 1.16–1.18). An increase was also seen when we looked specifically for the use of SSRIs in at least the first trimester (IRR = 1.18, 95 % CI 1.17–1.20). An increased IRR in the use of drugs for pulmonary disease was also seen when children were exposed to TCAs, but this was not statistically significant. However, in both groups our sample size was rather small. The effect size is modest and may also be confounded by maternal smoking.

**Conclusions:**

In utero exposure to SSRIs leads to a statistically significant increase in the use of drugs for pulmonary diseases, especially when exposure occurred during the first trimester of pregnancy. The increase in the use of drugs for pulmonary disease may also be related to other factors. Therefore, further study is recommended.

## Introduction

It has been estimated that up to 70 % of pregnant women experience some symptoms of depression, with 10–16 % of pregnant women fulfilling diagnostic criteria for a major depressive disorder [1, 2]. Drugs of choice during pregnancy for maternal depression are tricyclic antidepressants (TCAs) and fluoxetine, a serotonin reuptake inhibitor (SSRIs) [3]. Because of recent discussions about the efficacy of SSRIs in general, the use of SSRIs during pregnancy should be reconsidered [4]. However, the use of SSRIs has increased two- to fourfold in western society during the last decade and is therefore of importance for study [5, 6].

Most studies found no teratogenic effects for TCAs in general [7], although one Swedish study identified an association between maternal clomipramine use and congenital cardiovascular anomalies such as ventricular or atrial septal defects [8].

For SSRIs, studies have been published where paroxetine and maybe bupropion, sertraline and citalopram are at risk for cardiac congenital anomalies [9–15]. Paroxetine and also venlafaxin and fluoxetine, are at risk of pulmonary hypertension [16–19]. Further, SSRIs are possibly associated with an increased risk of omphalocele, anencephaly, and craniosynostosis [20]. Of interest is the study of Reis et al. where an increase in respiratory diagnosis (according to ICD-10 definitions) after in utero exposure to antidepressants was found [21]. In relation with studies about pulmonary hypertension associated with use of SSRIs it is worthwhile investigating further relations between in utero exposure of antidepressants and fetal lung development.

The respiratory tract develops after 4 weeks of conception from the ventral wall of the foregut. The epithelium of the internal lining of the respiratory system is of endodermal origin. Muscular and connective tissues are derived from the splanchic mesoderm. Up to the seventh prenatal month the bronchioles divide continuously into more and smaller canals (canalicular phase) and the vascular supply increases steadily. Respiration becomes possible when some of the cells of the cuboical respiratory bronchioles change into thin flat cells. The cells are intimately associated with numerous blood and lymph capillaries, and the surrounding spaces are now primitive alveoli. At the end of the sixth month type 2 alveolar cells produce surfactant, a phospholipid rich fluid capable of lowering surface tension at the air-alveolar interface [22].

Serotonin (5-HT) from pulmonary neuro-endocrine cells (PNECS) and serotonin cells in the raphe nucleus may have different effects on the developing respiratory tract. 5-HT modulates via the 5-HT_1a_ receptor, the respiratory rhythm generator (RRG) [23, 24]. Further, 5-HT modulates, probably via the 5-HT_1B_ receptor and regulated by serotonin transporters, pulmonary vasoconstriction in mice, rats, and hamsters [25, 26], and depression of the phrenic motor neurons [23]. The 5-HT_2A_-receptor is involved in the tonic firing of phrenic motor neurons [23]. Activation of the 5-HT_2B_-receptor leads to elevated respiratory activity via the pre-Bötzinger complex (PBC) [27]. Together with the 5-HT_2A_-receptor, the 5-HT_1B_ receptor is crucial for sustained spontaneous respiratory activity [27]. 5-HT from PNECS induces mechanical stretch of the lungs, which stimulates proliferation and maturation of fetal lung cells and the production of surfactant [28]. These findings together make SSRIs that enhance 5-HT action in the synaptic cleft, and may influence fetal lung function.

The other pharmacological class of antidepressants, TCAs, may also have an influence on the fetal development of the respiratory tract. TCA administration leads to elevated levels of norepinephrine. Administration of isoxsuprine, a β-adrenergic agonist, influences the production of surfactant [29, 30]. This is in contrast to an α-adrenergic receptor agonist (phenylephrine) [29]. Norepinephrine and other agonists may also be involved via adrenergic receptors in the activity of the RRG [31, 32]. These findings ensure that the possible influence of TCAs on the fetal development of the respiratory system cannot be ruled out.

The aim of this study was find out if in utero exposure of antidepressants might lead to enhanced use of drugs for pulmonary diseases. Therefore, we used as a proxy for developmental respiratory tract changes compared with non-exposure to antidepressants in particular, exposure of antidepressants during the 2nd and 3rd trimesters. The time periods are specific because of the involvement of antidepressants in the production of surfactant.

## Materials and methods

This study was performed with the IADB.nl-database, which contains pharmacy prescription data of an estimated population of 500,000 individuals from the Netherlands. Registration in the database is irrespective of health insurance and is considered representative of the general population. Each prescription record contains information on the date of dispensing, the quantity dispensed, the dose regimen, the number of days the prescription is valid, the prescribing physician, and the Anatomical Therapeutic Chemical (ATC) code. Each patient has a unique anonymous identifier; date of birth and gender are known. Because of the high level of patient–pharmacy commitment in the Netherlands, the medication records for each patient are virtually complete, except for OTC drugs and medication dispensed during hospitalization.

The data for this study were obtained from the ‘Pregnancy IADB’, which was extracted from the main IADB database. Children were selected by date of birth and the female person (15–50 years) with the same address code was considered to be the mother [33]. Because only the child’s birth date is known, the theoretical conception date was determined as the date of birth minus 273 days (i.e., 9 months). The database has been previously described [6, 34]. The data between 1995 and 2009 from the pregnancy database were used. Focusing on our study objective, we determined the relative risk of the use of drugs for pulmonary diseases in children after in utero antidepressant exposure.

### Study population, exposure and reference groups

We identified in the period 1995–2009, 35,546 children from 23,576 women, as some women gave birth to more than one child. The exposure to antidepressants was calculated during the three trimesters of pregnancy of each woman. Exposure was defined as the theoretical period of use: from dispensing date until the last day the prescription was valid. Two exposure groups were formed, including children of mothers exposed to TCAs (ATC code = N06AA; *n* = 67) and children of mothers exposed to selective serotonin release inhibitors (SSRIs; ATC code = N06AB; *n* = 436) during pregnancy. We excluded children who were exposed to any SSRI and any TCA (*n* = 10), because of the interchangeable effects of both classes of drugs. All prescriptions of TCAs with less than 0.5 DDDs per day were excluded (*n* = 58), because TCAs are used for treating neuropathic pain (which has not been associated with pulmonary diseases) at a lower dose than for treating depression, the disease that has been associated with asthma. The time of exposure to antidepressants was divided into the following periods: any time during pregnancy, only the first trimester, only the second and third trimesters combined, at least the first trimester, and at least the second and third trimesters combined. The reference group consisted of women who did not use any SSRIs or TCAs during pregnancy and during a period of 7 days before pregnancy (*n* = 35,033).

### Drugs for pulmonary diseases

Drugs for pulmonary diseases in children were used as a proxy for developmental respiratory tract changes in children as a result of antidepressant exposure in utero. The use of beta-2 selective sympathomimetics (ATC = R03CC, and R03AC), inhalation corticosteroids (ATC = R03BA), combined inhalation drugs (ATC = R03AK), and leucotriene antagonists (ATC = R03DC) in children were studied in the exposed groups and the reference group, and was regarded as a proxy for developmental respiratory tract changes in children if two or more prescriptions within 1 year were found in our database.

### Covariate analysis

From the literature we know that in utero exposure to antibiotics or benzodiazepines may lead to pulmonary diseases, and also maternal diabetes, maternal age of delivery and maternal (genetic) asthma, and may lead to pulmonary diseases in the offspring. The exposure to these factors at any time during pregnancy was considered a possible confounding factor (see Discussion section). Therefore, we tested with Chi-squared analysis for differences between the exposed groups (Table 1). Those factors that differed between the groups and significantly increased the IRR for use of drugs for pulmonary disease, will be included in our calculations for adjusting the crude IRR. We calculated the adjusted IRR with the formulas given in Rothman [35].

**Table 1:** Group characteristics and possible confounding factors

	Reference group (%)	Exposed to SSRIs (%)	Exposed to TCA (%)	*p* value
Total	35,033 (100 %)	436 (100 %)	67 (100 %)	
Maternal use of antibiotics	7,027 (20.1 %)	115 (26.4 %)	17 (25.4 %)	0.005
Maternal use of benzodiazepines	883 (2.5 %)	121 (27.8 %)	19 (28.4 %)	<0.001
Maternal age > 30 years at delivery	15,585 (44.5 %)	244 (56.0 %)	34 (50.7 %)	<0.001
Maternal use of insulin	269 (0.8 %)	2 (0.5 %)	1 (1.5 %)	0.896
Maternal use of drugs for pulmonary diseases	1,432 (4.1 %)	21 (4.8 %)	6 (9.0 %)	0.285

SSRIs = serotonin reuptake inhibitors, TCAs = tricyclic antidepressants

### Analysis

The calculated day of conception was chosen as the starting point to identify in which periods the children were exposed. The day of birth of the children was chosen as a starting point for the follow-up. The incidence rate (IR) of drugs for pulmonary diseases used in the defined exposure groups was calculated as the number of incident cases (drugs for users with pulmonary diseases) divided by the time at risk (in years). The time at risk was measured from the day of birth until either the first prescription date, or the last known date of the child in the database, or the end of the study period, whichever occurred first. The exposure groups and reference group were compared and the incidence risk ratio (IRR) and 95 % confidence interval (CI) were calculated according to Rothman [35].

## Results

From the 35,400 pregnancies in our population, 36,323 children were born. Exposure to an SSRI anytime in pregnancy occurred in 436 children. Paroxetine was the most commonly prescribed SSRI (*n* = 266), followed by fluoxetine (*n* = 111), citalopram (*n* = 91), fluvoxamine (*n* = 70), sertraline (*n* = 34), and escitalopram (*n* = 11) (the sum of users exceeds the number of SSRI users because of concomitant use of two or more SSRIs or change of SSRI during pregnancy). Exposure to a TCA anytime in pregnancy occurred in 67 children. The most commonly used TCA was clomipramine (*n* = 43), followed by amitriptyline (*n* = 31). In the period 1995–2009, a total of 35,033 children were not exposed to antidepressant medication.

We found a significant increase in the use of drugs for pulmonary disease in children who were exposed to SSRIs any time in utero (incidence risk ratio = 1.17; 95 % confidence interval 1.16–1.18; Table 2). We also found an increased risk when exposure was at least in the first trimester (IRR = 1.18, 95 % CI 1.17–1.20). The increase, although not statistically significant, was also found for TCAs where IRR was 1.07 (95 % confidence interval 0.96–1.19; Table 2). The size of the crude IRR of the use of drugs for pulmonary disease due to antidepressant exposure was for all periods during pregnancy more or less the same. However, adjusted for concomitant antibiotic exposure, only the use of SSRIs, at any time during the pregnancy and at least during the first trimester, leads to a statistically significant increase in the use of drugs for pulmonary disease.

**Table 2:** The use of drugs for pulmonary diseases after in utero exposure to SSRIs or TCAs

Group	Pregnancy period	Children exposed	Use of drugs for pulmonary disease	Time at risk (years)	IR (years)	IRR (95 % CI)	IRR (95 % CI) aadjusted^a^
Reference	Anytime	35,033	6,722	13,244.12	0.508	1	1
Exposed to SSRIs	Anytime	436	83	136	0.61	1.20 (0.97– 1.49)	1.17 (1.16– 1.18)
	Only 1st trimester	163	30	56.7	0.53	1.04 (0.73– 1.49)	1.03 (0.98– 1.09)
	Only 2nd and 3rd trimester	26	3	4.72	0.64	1.26 (0.41– 3.91)	^b^
	At least 1st trimester	374	74	121.88	0.61	1.20 (0.96– 1.51)	1.18 (1.17–1.2)
	At least 2nd and 3rd trimester	195	39	59.11	0.66	1.30 (0.95– 1.78)	^b^
Exposed to TCAs	Anytime	67	12	21.47	0.56	1.10 (0.63– 1.94)	1.07 (0.96– 1.19)
	Only 1st trimester	31	8	13.47	0.59	1.16 (0.58– 2.32)	^b^
	Only 2nd and 3rd trimester	3	0				
	At least 1st trimester	57	11	20.93	0.53	1.04 (0.58– 1.88)	^b^
	At least 2nd and 3rd trimester	24	3	7.46	0.4	0.79 (0.25– 2.44)	^b^

IR = incidence risk, IRR = incidence risk ratio

^a^Adjusted for maternal antibiotic use

^b^Insufficient number of cases for adjustment

Some possible confounding factors, like in utero exposure to antibiotics and in utero exposure to insulin, showed a significant increase in the use of drugs for pulmonary disease (Table 3). Therefore, differences between the groups exposed to antidepressants combined with the increase due to confounding factors, leads to adjustment of the crude IRR for maternal use of antibiotics. After adjustment, the effect of the SSRIs on children exposed to antidepressants any time during pregnancy and at least during the first trimester turned into a significant increase in the use of drugs for pulmonary disease (IRR = 1.17, 95 % CI 1.16–1.18; 95 % CI 1.17–1.20). Patient numbers of other time-frames of the pregnancy were in general too small for adjustments of the crude IRR.

**Table 3:** The use of drugs for pulmonary diseases after in utero exposure to possible confounders

Group	Children exposed	Use of drugs for pulmonary disease	Time at risk (years)	I (years)	IRR	IRR (95 % CI)
Reference	35,536	6,807	13,244.12	0.51	1	1
In utero exposure to antibiotics	7,159	1,672	3,030.12	0.55	1.08	1.08 (1.02–1.14)
in utero exposure to benzodiazepines	1,027	227	484.45	0.47	0.92	0.92 (0.81–1.05)
Children with a mother aged > 30 at delivery	15,863	2,877	5435.73	0.53	1.04	1.04 (1.00–1.09)
In utero exposure to insulin	272	49	67.15	0.73	1.43	1.43 (1.08–1.89)
In utero exposure to drugs for pulmonary disease	1,459	487	866.65	0.56	1.1	1.10 (1.00–1.21)

## Discussion

We hypothesized that antidepressants influence the fetal lung development because of the involvement of 5-HT and noradrenergic receptors in the development of the mammalian respiratory rhythm generator and the production of surfactant. We tested this hypothesis by counting the number of prescriptions of drugs for pulmonary diseases of children who were exposed in utero to antidepressants as a proxy for developmental respiratory tract changes. We found a significant increase in the use of drugs for pulmonary disease after in utero exposure to SSRIs any time during pregnancy (IRR = 1.17, 95 % CI 1.16–1.18) after adjustment for in utero exposure to antibiotics. Also, an increased IRR of children exposed in utero to SSRIs during at least the first trimester was found (IRR = 1.18, 95 % CI 1.17–1.20). The increase was seen in all periods of pregnancy; however, probably because of our small sample size, statistical significance was only reached in the first trimester or at any time during pregnancy. We also found an increase in the use of drugs for pulmonary disease after in utero exposure to TCAs (IRR = 1.07, 95 % CI 0.96–1.19), also after adjustment of in utero exposure to antibiotics.

Our results are in line with other findings. Kozyrskyj et al. reported that maternal distress, using prescriptions of antidepressants and anxiolytics, leads in 8.3 % of children exposed to antidepressants to asthmatic diseases [36]. The question now arises, what is the contribution of antidepressants compared with anxiolytics in the study of Kozyrskyj et al., because he took antidepressants and anxiolytics together in his analysis, whereas we found an increased risk of SSRIs alone. Recently, Reis and Kallen reported an increase in neonatal respiratory diagnoses after in utero exposure to antidepressants [21]. Both studies suggest an increase in the use of drugs for pulmonary disease due to in utero exposure to antidepressants.

Drug-related factors that might be involved in the use of drugs for pulmonary diseases in childhood are gestational diabetes (use of insulin), intrauterine exposure to sympathomimetics (maternal use of drugs for pulmonary diseases), maternal antibiotic use during pregnancy, and maternal use of benzodiazepines [37, 38]. These factors were used for a co-variate analysis in our database. Another finding that depression itself is related to the occurrence of asthma via neuroimmunomodulatory pathways led to us investigating the role of benzodiazepines, which could serve as a surrogate drug for depression and anxiety [36]. Further to overcome confounding by indication, we excluded mothers who use less than 0.5 DDD of TCA, which is probably used for neuropathic pain. Because other factors were not different between the exposed and the unexposed groups, we did not correct for maternal use of insulin, and maternal use of drugs for pulmonary disease.

Of importance is that maternal and/or paternal smoking in the presence of their children could not be detected in our database-oriented study. Therefore, further research is mandatory. It is well known that the incidence of smoking among psychiatric patients is higher than in a nonpsychiatric population [39]. The negative effects on the respiratory system of maternal smoking may lead to increased use of drugs for pulmonary diseases in mother and child. Independently, maternal pulmonary diseases affect fetal respiratory tract development [37, 38]. It was also shown that maternal asthma is statistically significantly associated with congenital anomalies of the respiratory system, which might further increase the use of drugs for pulmonary diseases [40]. We found that maternal asthma expressed as the use of drugs for pulmonary disease did not lead to an increase in the use of drugs for pulmonary disease in the child, however slightly statistically nonsignificant. Because there was no difference between the exposed and the non-exposed group in the use of maternal drugs for pulmonary disease, we did not adjust for maternal use of drugs for pulmonary disease.

Remarkable is our finding that despite pharmacological support for developmental changes of the respiratory tract due to in utero exposure of antidepressants, we did not find differences between the different periods of pregnancy and the class of antidepressants and the increase in the use of drugs for pulmonary diseases in children. Probably, the low number of children exposed in only the second and third trimesters is the reason for this.

The strength of our study is that we searched for and found a pharmacological explanation for the possible involvement of antidepressants in the developing fetus, and that we could test this hypothesis with the use of prescription data from mother and child. With this approach, using prescriptions of drugs as a proxy for diseases or influence on developmental processes, we could also correct for possible confounding variables like in utero exposure to antibiotics. However, there are also some limitations in using an administrative prescription database because we do not know whether the drugs were actually taken. If the women had not been compliant, our results would have been an underestimation of the real effect. Another limitation is that over-the-counter drugs (OTC) are lacking. These drugs may act as a confounder for our results. SSRIs and TCAs are prescribed for treating depression, but also for treating anxiety and personality disorders. These diseases or the behavioural changes of the mothers may be associated with the use of drugs for pulmonary diseases. We do not have information about the indication for the prescribed drugs, and therefore confounding by indication is still possible. Only randomized controlled follow-up studies could prevent this form of confounding.

Tricyclic antidepressants and serotonin reuptake inhibitors also bind to receptors that were not investigated (muscarine-M-3-receptor, histamine-H-1-receptor), but the function of these receptors in the ontogeny of the respiratory tract is still unclear.
